# Influence of Mothers

**Published:** 1878-07

**Authors:** 


### INFLUENCE OF MOTHERS.

JOHN RANDOLPH never ceased, till his dying day, to remember with unutterable affection the pious care of his mother, in teaching him to kneel at her side, and, with his little hands pressed together, and raised upwards, to repeat, in slow and measured accents, the pattern prayer.

" My mother," said Mr. Benton, not long before he died, " asked me not to drink liquor, and I never did. She desired me at another time to avoid gaming, and I never knew a card. She hoped I would not use tobacco, and it never passed my lips."

